# Adaptation and validation of Spanish version of the Inpatient Dignity Scale in hospitalized patients: a psychometric study

**DOI:** 10.1016/j.ijnsa.2025.100423

**Published:** 2025-09-13

**Authors:** Juan Roldan-Merino, Carmen Jerez-Molina, Olga Mestres-Soler, Lucia Muñoz-Narbona, Montserrat Gutiérrez-Juarez, Ainoa Biurrun-Garrido, Jéssica Gutiérrez-Martínez, Jurema Lopez-Monreal, Clara Expósito-Guanter, Martí Boix-Coll, Lucia Peñarrubia-San-Florencio, Ramon Mir-Abellan

**Affiliations:** a Campus Docent Sant Joan de Deu, Universitat de Vic-Universitat Central de Catalunya (UVIC-UCC), C/ Sant Benito Menni, 18-20, 08830 Sant Boi de Llobregat, Barcelona, Spain; b Mental Health, Psychosocial and Complex Nursing Care Research Group-2021 SGR 01083, Spain; c Research Group DAFNIs. Campus Docent Sant Joan de Deu, Universitat de Vic-Universitat Central de Catalunya (UVIC-UCC), C/ Sant Benito Menni, 18-20, 08830 Sant Boi de Llobregat, Barcelona, Spain; d Vall d'Hebron University Hospital, Pg. de la Vall d'Hebron, 119, Horta-Guinardó, 08035, Barcelona, Spain; e Associate Nursing Department Professor UAB, Barcelona, Spain; f Collaborator professor Nursing Department. Campus Docent Sant Joan de Deu, Universitat de Vic-Universitat Central de Catalunya (UVIC-UCC), C/ Sant Benito Menni, 18-20, 08830 Sant Boi de Llobregat, Barcelona, Spain; g Multidisciplinary Nursing Research Group, VHIR Vall d'Hebron Institute of Research, Barcelona, Spain; h Clinical Research Coordinator, Germans Trias i Pujol Research Institute (IGTP), Campus Can Ruti. Carretera de Can Ruti, Camí de les Escoles s/n. 08916 Badalona, Barcelona, Spain; i Universitat Autònoma de Barcelona (UAB), Barcelona, Spain; j NURECARE Research Group, Germans Trias i Pujol Research Institute (IGTP) i Germans Trias i Pujol University Hospital, 08916 Badalona, Spain; k Área de Conocimiento. Hospital San Juan de Dios de Barcelona, Sant Joan de Déu, 2, 08950 Esplugues de Llobregat, Spain; l Grupo Clínico Asociados “Investigación en enfermería” Instituto de Investigación San Juan de Dios (IRSJD), Santa Rosa 39-57, 08950, Esplugues de Llobregat, (Codi ROR: 00gy2ar74), Spain; m Infermera Servei Endoscòpies. Gestora de casos del Programa de Cribatge de Càncer Colorectal, Parc Sanitari Sant Joan de Déu, Sant Boi de Llobregat, Barcelona, Spain; n Infermers de Recerca. Germans Trias i Pujol Research Institute (IGTP). Campus Can Ruti. Carretera de Can Ruti, Camí de les Escoles s/n. 08916 Badalona, Barcelona, Spain; o Servicio de atención paliativa pediátrica y paciente con enfermedad crónica compleja, C2P2. Hospital San Juan de Dios Barcelona, Sant Joan de Déu, 2, 08950 Esplugues de Llobregat, Spain.; p Grupo Clínico Asociados: "Cronicidad y cuidados paliativos pediátricos" Instituto de Investigación San Juan de Dios (IRSJD), Santa Rosa 39-57, 08950, Esplugues de Llobregat (Codi ROR: 00gy2ar74), Spain; q Parc Sanitari Sant Joan de Déu. Carrer del Dr. Antoni Pujadas, 42, 08830. Sant Boi de Llobregat, Barcelona, Spain; r Gestor del Conocimiento del Parc Sanitari Sant Joan de Déu. Carrer del Dr. Antoni Pujadas, 42, 08830. Sant Boi de Llobregat, Barcelona, Spain

**Keywords:** Respect, Hospitalization, Psychometrics, Reproducibility of results, Nursing care, Patient-centered care

## Abstract

**Background:**

Maintaining dignity in hospitalized patients is fundamental in nursing care and healthcare quality. In Spain, there is no validated tool to quantitatively assess patient dignity during hospitalization. The Inpatient Dignity Scale has been developed and validated in other countries, but its cross-cultural adaptation and psychometric evaluation in Spanish have not yet been conducted.

**Objective:**

To perform cross-cultural adaptation and psychometric validation of the Inpatient Dignity Scale in Spanish, evaluating its factorial structure, reliability, and validity.

**Methods:**

A methodological study was conducted in two phases: (1) translation and cross-cultural adaptation following international standards, and (2) psychometric validation through confirmatory factor analysis and assessment of internal consistency using Cronbach's alpha (α) and McDonald's omega (Ω) coefficients. The sample included 553 hospitalized patients from four hospitals in Barcelona, Spain.

**Results:**

The confirmatory factor analysis confirmed the original four-factor structure of the Inpatient Dignity Scale, maintaining the dimensions of respect as a human being, respect for personal feelings and time, respect for privacy, and respect for autonomy. Model fit indices were adequate (χ²/df = 5.7 for expectations, 4.3 for satisfaction; CFI ≥ 0.90; RMSEA ≤ 0.08). Internal consistency was high across all dimensions (α ≥ 0.88, Ω ≥ 0.92), indicating robust reliability.

**Conclusions:**

The Spanish version of the Inpatient Dignity Scale in Spanish is a valid and reliable instrument for assessing expectations and satisfaction with dignity in hospitalized patients. Its use can facilitate research on dignity in healthcare and contribute to improving patient-centered care.

## Highlights

• **What is already known**
• Maintaining patient dignity is a core component of nursing care and a marker of healthcare quality.
• The Inpatient Dignity Scale is a validated instrument in several countries for assessing dignity in hospitalized patients.
• There is a lack of validated tools in Spanish to quantitatively measure patient dignity during hospitalization.
• **What this paper adds**
• This study provides the first validated Spanish version of the Inpatient Dignity Scale using rigorous cross-cultural adaptation and psychometric evaluation.
• The Spanish version confirmed the original four-factor structure and demonstrated strong internal consistency and construct validity.
• This tool enables researchers and clinicians in Spanish-speaking contexts to systematically assess patient dignity and enhance dignity-conserving care practices.

## 1 Introduction

Human dignity is a fundamental pillar of human rights, with a universal scope that transcends borders (Nations, 1948).

The second meaning is status or meritocratic dignity, associated with social recognition (Sinnot, 2007). Over time, this notion has become associated with wealth and social status, reflecting society’s influence on its definition (Düwell et al., 2014).

Finally, patient dignity is an essential value, and nursing professionals play a crucial role in its preservation (Baillie, 2009; Shotton and Seedhouse, 1998). Care dignity refers to ensuring that each patient is treated with respect, promoting their autonomy and well-being. This concept has been widely recognized as a fundamental right, endorsed by international organizations such as the World Health Organization (WHO, 1978). In this context, care dignity encompasses not only the ethical treatment of patients but also the establishment of a care environment that ensures privacy, safety, a sense of being valued and respected, effective communication, and active participation in decision making. These dimensions are central to recent international efforts aimed at integrating dignity conserving practices such as dignity therapy and routine privacy checks into routine inpatient care (Chochinov, 2007; Hadler et al., 2024).

Preserving dignity in the hospital setting is a constant challenge for healthcare professionals, as factors such as care overload and resource shortages may hinder its effective implementation (Guerra-Paiva et al., 2023; Stephen Ekpenyong et al., 2021).

In nursing, care dignity is a key element of professional ethics (Simões and Sapeta, 2019). Baillie (2009) has reinforced its relevance in care quality, establishing that ensuring patient dignity in healthcare is now a professional duty.

Additionally, dignity in healthcare refers to how individuals feel, think, and behave concerning their own value and that of others (Saxena and Hanna, 2015). It is, therefore, a humanistic concept based on respect for human integrity and personal beliefs (Kadivar et al., 2018). Moreover, patients with an intrinsic sense of dignity maintain a positive perception of themselves and their illness, highlighting the need for care strategies that consider dignity as an inherent quality (Rodríguez-Prat et al., 2016).

To date, qualitative studies involving small cohorts of hospitalized patients have been published, highlighting key aspects such as respect for confidentiality, privacy, and participation in treatment decisions. In the context of end-of-life care, patients have also described dignity as a way of being and behaving in the face of illness (Du et al., 2024; Mabega et al., 2024; Martí-García et al., 2023; Shakwane, 2023).

Recent systematic reviews based on patient experiences during hospitalization consistently report that privacy, autonomy, respect, and compassionate relationships with healthcare professionals are essential to preserving patient dignity in hospital settings.

Dignity is a fundamental attribute of human behavior, and its loss has been associated with increased patient suffering. Demographic, clinical, and psychological factors may influence how patients experience and perceive dignity.

Furthermore, these reviews emphasize a lack of interventional studies in this field, identifying a critical gap in the evidence base that should be addressed through well-designed research with robust outcome measurements (Eduardo Pereira Dutra et al., 2022; Fuseini et al., 2022)

Given the fundamental role of dignity in healthcare, various instruments have been developed to assess it from different perspectives. These include patient perception, healthcare staff perspectives, and the impact of the hospital environment on the care experience (Lam et al., 2022; Li et al., 2018; Rial et al., 2006; Wong et al., 2015; Fuseini, Mohebbi, et al., 2023).

These instruments have facilitated the analysis of key aspects such as respect, autonomy, privacy, and communication in the care relationship, providing valuable insights for improving care quality. However, most of these tools have been developed and validated in different sociocultural contexts, which may limit their applicability in other healthcare settings.

Although some instruments have been validated in Spanish, their use has been primarily focused on specific populations, such as cancer patients (Arias-Rojas et al., 2022; Rullán et al., 2015), where dignity perception is particularly relevant due to their emotional and physical vulnerability. Additionally, other instruments validated in Spanish assess patient experiences during hospitalization, evaluating aspects such as satisfaction with received care or communication with healthcare professionals. However, these tools do not specifically address dignity in received care, limiting their ability to comprehensively capture this concept within the hospital setting (Rodríguez-Martín et al., 2019).

In the Spanish context, the scientific literature reveals a lack of quantitative tools designed to assess care dignity in hospitalized patients. This lack of validated instruments contrasts with the availability of internationally recognized scales, such as the Inpatient Dignity Scale (Ota et al., 2019), which has been developed and implemented in countries such as Japan and the United Kingdom. The Inpatient Dignity Scale has proven to be a valuable resource for assessing dignity in hospital care, providing key insights to improve care quality and ensure a more humanized approach to patient care.

Given the importance of culturally adapted and validated measurement tools, the present study aims to conduct the cross-cultural adaptation and psychometric evaluation of the Inpatient Dignity Scale in the Spanish context (Ota et al., 2019). Validating this instrument will allow for precise and culturally relevant measurements of dignity perception in hospital care, facilitating its implementation in both research studies and clinical practice. This will contribute to improving care quality by promoting interventions aimed at ensuring respect, autonomy, and the well-being of hospitalized patients.

## 2 Methods

### 2.1 Study design

A psychometric study with a methodological approach was conducted to adapt and validate the Spanish version of the Inpatient Dignity Scale. The process was carried out in two stages: first, the scale was translated and culturally adapted into Spanish, and subsequently, its psychometric properties were analyzed to assess its validity and reliability in this new version.

### 2.2 Study setting and sample

The psychometric properties of the instrument were analyzed in a sample of hospitalized patients from four hospitals in the province of Barcelona: Hospital Universitari Germans Trias i Pujol (Badalona), Hospital Maternoinfantil de Sant Joan de Déu (Barcelona), Hospital Vall d’Hebron (Barcelona), and Parc Sanitari Sant Joan de Déu (Sant Boi de Llobregat).

The sample size was determined following various recommendations from experts in psychometric analysis. Floyd and Widaman (Floyd and Widaman, 1995) suggest using between 5 and 10 participants per variable in Confirmatory Factor Analysis, while Comrey (2013) states that a sample of 500 participants is appropriate for psychometric analyses. Considering these recommendations, a minimum sample of 500 participants was established, with at least 100 participants per hospital, ensuring a solid foundation for the analysis.

A non-probabilistic convenience sampling method was used. Inclusion criteria were being a hospitalized patient in a medical-surgical unit of the participating hospitals, being 18 years or older, and voluntarily agreeing to participate in the study. Patients admitted to intensive care units, as well as those with cognitive impairment or psychiatric disorders that could affect their ability to autonomously and comprehensibly complete the questionnaire, were excluded.

### 2.3 Data collection

The data collection process was conducted between January and December 2024. A structured form was designed to collect information, consisting of two sections: the first gathered sociodemographic data and type of admission, while the second included the Spanish version of the Inpatient Dignity Scale. Both sections were digitized and managed through the REDCap platform (Harris, 2012), ensuring a secure and standardized data recording process.

In all participating hospitals, efforts were made to administer the questionnaires at appropriate times to ensure an optimal environment for completion, minimizing interruptions and facilitating patient participation.

### 2.4 Variables and source of information

Variables collected from hospitalized patients included age, gender, nationality, marital status, family composition, religion, and type of hospital admission. Additionally, variables related to the Inpatient Dignity Scale were included. These variables were selected to provide a comprehensive understanding of the factors influencing patient dignity and well-being during hospitalization.

The Inpatient Dignity Scale (Ota et al., 2019) is an instrument designed to assess the expectations and satisfaction of hospitalized patients regarding dignity in healthcare. The final version of the scale consists of two subscales: one for evaluating expectations and another for satisfaction, each comprising 21 items grouped into four dimensions that reflect key aspects of dignity preservation during hospitalization.

All items are rated on a 5-point ordinal scale. In the expectations subscale, the response options are: 1= no expectations, 2= not very high expectations, 3= moderately high expectations, 4= high expectations, and 5= very high expectations. In the satisfaction subscale, the response options are: 1= very dissatisfied, 2= somewhat dissatisfied, 3= moderately satisfied, 4= satisfied, and 5= very satisfied.

The internal consistency of the original version of the scale, assessed using Cronbach’s alpha, showed reliability coefficients above 0.70 in each of the four dimensions, both in the expectations and satisfaction subscales, as well as in the overall scale score. These results confirm the robustness and reliability of the Inpatient Dignity Scale as a psychometric tool for assessing dignity in hospitalized patients.

The item scoring and distribution within each factor for the expectations and satisfaction subscales are presented in Table 1.

**Table 1:** Item distribution within each factor for the expectations and satisfaction subscales of the Inpatient Dignity Scale.

**Dimension**	**For expectation of dignity**		**For satisfaction with dignity**	
	Items	Min–Max	Items	Min–Max
F1. Respect as a human being	1, 2, 3, 4, 5, and 6	6−30	1, 2, 3, 4, 5, and 6	6−30
F2. Respect for personal feeling and time	7, 8, 9, 13 and 14	5–25	Item 8, 9, 10, 14, 15, 16, 17 and 18	8−40
F3. Respect for privacy	19, 20 and 21	3−15	19 and 21	2−10
F4. Respect for autonomy	11 and 12	2−10	11 and 12	2−10
Total item	For expectation of dignity: items 10,15,16,17, and 18 should be excluded.	16−80	For satisfaction with dignity: items 7,13, and 20 should be excluded.	18−90

### 2.5 Procedure

The Inpatient Dignity Scale was translated into Spanish through a rigorous process aimed at ensuring linguistic, conceptual, and cultural equivalence between the original and target versions (Beaton et al., 2000). This approach aims to preserve the instrument’s validity and reliability across different cultural contexts.

First, authorization was obtained from the original scale’s authors for its adaptation into Spanish. The initial translation into Spanish was carried out by two independent translators who were unfamiliar with the scale and the study objectives, to ensure a neutral and accurate translation.

A panel of experts, consisting of seven nurses with experience in ethics, clinical practice, and psychometrics, was assembled to assess the semantic equivalence of the translated version. The panel systematically reviewed the meaning and clarity of the items in both versions, ensuring that the conceptual integrity of the original scale was preserved. All items were included in the Spanish version, and semantic equivalence was established through expert consensus. Unanimous approval from all panel members was required for item inclusion, achieving consensus in the first Spanish version of the scale.

Subsequently, the Spanish version was back-translated into the original language by two native English-speaking translators to verify its correspondence with the original scale. The research team thoroughly reviewed the back-translation and compared it with the original version to ensure accuracy and conceptual consistency.

During the cross-cultural adaptation process, a discussion was held regarding the potential replacement of the term P/N (Physicians / Nurses) with P/S (healthcare personnel) to promote a more inclusive terminology. However, after careful consideration and expert consensus, it was decided to retain the original *“M/E (médico/Enfermera)”* terminology to ensure fidelity to the clinical context of the instrument.

Several modifications were made to specific items to enhance semantic and cultural equivalence. In Item 1, the wording was adapted to improve clarity and applicability, resulting in the final version: *"M/E me tratan y me cuidan como a un ser humano y no como a un objeto."* In Item 9, the original phrase "(P/N) greet me first when they see me in the hospital" was modified by omitting the term hospital, as it was considered unnecessary in the general clinical context. The final version was *"M/E me saludan cuando me atienden".* For Item 10, the phrase "(P/N) treat my pain promptly" was reviewed, particularly in relation to the translation of promptly. It was agreed that the expression *"de manera adecuada”* better captured the intended meaning rather than *"inmediata o rápida,"* leading to the final version: " *"M/E tratan mi dolor de manera adecuada."* Item 13 generated some debate regarding the most appropriate wording to reflect patient preference without introducing bias. The original phrase "(N) of my gender give me care" was ultimately adapted to use the term *"prefiero"* as a more suitable option, resulting in the final version: "*(N) Prefiero que enfermeras de mi mismo sexo me atiendan”*.

Once the final version of the questionnaire was established, a content validity assessment was conducted to evaluate the clarity, relevance, and representativeness of the items. A panel of experts independently rated each item on a four-point scale (1= not relevant, 2= somewhat relevant, 3= relevant, and 4= very relevant). All items were rated as either 3 (relevant) or 4 (very relevant) by all experts. All items were rated as either 3 or 4 by all experts. Based on these results, the item-level content validity index (I-CVI) was 1.00 for all items, and the scale-level content validity index (S-CVI) was also 1.00, indicating unanimous agreement on the relevance of all items.

The panel of experts included one midwife, two nurses working in medical-surgical units, one mental health nurse, and three internal medicine nurses, all with clinical experience relevant to the construct being assessed.

In addition, during the cognitive pretesting phase, verbal feedback was obtained from hospitalized patients to ensure the clarity and cultural appropriateness of the adapted items. Although this feedback was not recorded or formally documented, no comprehension difficulties were noted, and all participants expressed that the items were clear and acceptable.

Subsequently, a pilot test was conducted with 25 hospitalized patients to assess the overall comprehension of the items and estimate the time required to complete the scale, which was approximately 15 min. Based on the pilot test, minor modifications to the format were made to facilitate questionnaire completion.

Table 2 presents the semantic equivalence of the items in English and their corresponding adaptation into Spanish, ensuring linguistic and conceptual consistency in the translation process.

**Table 2:** Semantic equivalence of items from English to Spanish after psychometric validation.

	**English**	**Spanish**
1	(P/N) treat and care for me as a living human being rather than an object.	(M/E) me tratan y me cuidan como a un ser humano y no como un objeto.
2	(P/N) maintain eye contact with me while talking.	(M/E) mantienen contacto visual conmigo mientras me hablan
3	(P/N) respect me as a human being.	(M/E) me respetan como un ser humano.
4	(P/N) listen to me attentively.	M/E) me escuchan atentamente.
5	(P/N) always use polite language.	M/E) siempre utilizan un lenguaje educado.
6	(P/N) are polite to my family as well as to me.	(M/E) son educados tanto con mi familia como conmigo.
7	(P/N) talk to me at my eye level by sitting on a chair or bending.	(M/E) me hablan a la altura de los ojos aproximándose a mí.
8	(P/N) give my needs or expectations priority in their everyday practice.	(M/E) priorizan mis necesidades o expectativas en su práctica diaria.
9	(P/N) greet me first when they see me in the hospital.	(M/E) me saludan cuando me atienden.
10	(P/N) treat my pain promptly	(M/E) tratan mi dolor de manera adecuada.
11	(P/N) let me participate in the decision-making processes regarding my own treatment choices.	(M/E) me dejan participar en los procesos de toma de decisiones sobre mis propias opciones de tratamiento.
12	(P/N) offer different choices so I can decide on my treatment.	(M/E) me ofrecen diferentes opciones para que pueda decidir mi tratamiento.
13	(N) of my gender give me care	(E) Prefiero que enfermeras/os de mí mismo sexo me atiendan.
14	(P/N) understand my suffering and sympathize with me.	(M/E) comprenden mi sufrimiento y empatizan conmigo.
15	(P/N) are always cheerful to me.	(M/E) siempre me animan
16	(P/N) talk to me privately about my issues without allowing others to hear.	(M/E) Hablan conmigo en privado sobre mis problemas sin permitir que otros lo oigan.
17	(P/N) keep me protected with covering or clothing while providing medical treatment or nursing care.	(M/E) me protegen con cobertores o ropa mientras me proporcionan tratamiento médico o cuidados de enfermería.
18	(P/N) draw the bedside curtain or shut the door to maintain privacy during medical treatment or nursing care.	(M/E) corren la cortina de la cabecera o cierran la puerta para mantener la intimidad durante el tratamiento médico o los cuidados de enfermería
19	(P/N) share my information with other members of the health-care team if necessary.	(M/E) comparten mi información con otros miembros del equipo sanitario si es necesario
20	(P/N) do not disclose my sensitive information, such as family issues, to health-care workers other than my own physicians and nurses.	(M/E) no revelan mi información sensible, como asuntos familiares, a personal sanitario que no sean mis propios médicos y enfermeras.
21	(P/N) do not collect information that is unnecessary for my medical treatment or nursing care.	(M/E) no recogen información innecesaria para mi tratamiento médico o mis cuidados de enfermería
	**(N):** Nurses; **(P/N):** Physicians / Nurses	**(E):** Enfermera; **(M/E):** Médico / Enfermera

Note 1. For expectation of dignity: items 10,15,16,17, and 18 should; and for satisfaction with dignity: items 7,13, and 20 should be excluded.

## 3 Data analysis

### 3.1 Construct validity

For data analysis, the SPSS statistical package, version 28.0 for Windows (SPSS Inc., Chicago, IL, USA), was used, and confirmatory factor analysis was conducted using EQS, version 6.3 (Bentler RM and Wu EJC, 2016). To assess construct validity, a confirmatory factor analysis was performed using the generalized least squares method with a polychoric correlation matrix, a recommended approach for ordinal data analysis as it provides more robust parameter estimates compared to maximum likelihood estimation, which requires strict adherence to multivariate normality assumptions (Rial et al., 2006). Model fit was evaluated using multiple indices commonly reported in the structural equation modeling literature. First, the standardized chi-square (χ²/df) was calculated as the ratio between the χ² statistic and the degrees of freedom (df), considering values between 2 and 6 as indicative of an adequate model fit (Hu and Bentler, 1999). Additionally, the Comparative Fit Index (CFI), Goodness of Fit Index (GFI), and Adjusted Goodness of Fit Index (AGFI) were analyzed, establishing a threshold of ≥0.90 as an indicator of satisfactory model fit. The Root Mean Square Residual (RMR) and the Root Mean Square Residual Standardized (RMSR) were also examined, considering values ≤0.08 as indicative of good model parsimony. Lastly, the Root Mean Square Error of Approximation (RMSEA) was included, interpreting values ≤ 0.08 as evidence of an acceptable model fit, complemented by 90 % confidence intervals to assess the precision of the estimation (Brown, 2015; Comrey, 2013; Rial et al., 2006). The interpretation of these indices followed methodological standards established in the literature on instrument validation and confirmatory factor analysis, ensuring the robustness of the proposed model and its empirical adequacy. We also examine factor loadings (≥0.30 are deemed acceptable (Mulaik et al., 1989).

Construct validity was assessed through confirmatory factor analysis (CFA). To evaluate convergent and discriminant validity, Composite Reliability (CR) and Average Variance Extracted (AVE) were calculated for each latent factor. Convergent validity was considered adequate when AVE ≥ 0.50. Discriminant validity was assessed using the Fornell–Larcker criterion, by comparing the square root of each factor’s AVE with the inter-factor correlations.

### 3.2 Reliability

To evaluate the internal consistency of the instrument at both the overall level and for each specific factor, reliability was assessed using Cronbach’s Alpha and McDonald's Omega coefficients. Cronbach’s Alpha is a widely used measure that estimates the internal consistency of a scale based on the average correlation between items; however, its accuracy can be influenced by the number of items within a factor. To address this limitation, McDonald's Omega was also calculated, as it provides a more robust estimate by considering the contribution of individual factor loadings and correcting potential underestimations associated with Cronbach’s Alpha. A reliability threshold of ≥ 0.70 was considered indicative of acceptable internal consistency, following established methodological guidelines (Cronbach, 1951; Hayes and Coutts, 2020). These indices allowed for a comprehensive assessment of the scale’s reliability, ensuring that the instrument exhibited adequate internal coherence across all evaluated dimensions.

### 3.3 Ethical considerations

This study received approval from the following Research Ethics Committees (CEIC): Fundanción de Investigación Sant Joan de Déu (CEIC - PIC-133-21), Hospital Universitari Germans Trias i Pujol (CEIC - PIC-133-21), and the Research Ethics Committee of Hospital Universitari Vall d’Hebron (PR(AG)96/2023). The study was conducted in accordance with the principles of the Declaration of Helsinki and the General Data Protection Regulation (GDPR) of the European Union (“Issue Information ‐ Declaration of Helsinki”, 2023).

Prior to participation, all individuals were informed about the study’s objectives, procedures, and their rights, emphasizing the voluntary nature of participation and the guarantee of confidentiality. Participants received the Patient Information Sheet (FIP), which outlined the study’s purpose, data management process, and privacy protection measures. After reviewing the FIP and agreeing to participate, written Informed Consent (CI) was obtained from all participants.

Subsequently, questionnaires were self-administered via REDCap, a secure platform for data collection and management in clinical research (Harris, 2012). For patients who experienced difficulties completing the digital questionnaire through REDCap, a paper version was provided to facilitate their participation. These responses were later manually entered into the platform.

All data were securely stored on the servers of the Fundación de Investigación Sant Joan de Déu, with restricted access limited to the research team. No personally identifiable information was collected, nor was any data shared with third parties.

## 4 Results

### 4.1 Participant characteristics

The sociodemographic characteristics of the study population are presented in Table 3. The sample included 553 participants, with a mean age of 57.6 years (SD= 18.5). The majority of participants were attended at Parc Sanitari Sant Joan de Déu (36.5 %), followed by Hospital Vall d’Hebron (24.1 %) and Hospital Universitari Germans Trias I Pujol (23.1 %).

**Table 3:** Sociodemographic characteristics of the study population (*n* = 553).

	n	%
**Age: Mean (SD)**	57,6 (18,5)	
**Healthcare center**		
Parc Sanitari Sant Joan de Déu	202	36,5
Hospital Universitari Vall d’Hebron	133	24,1
Hospital Universitari Germans Trias i Pujol	128	23,1
Hospital Maternoinfantil Sant Joan de Déu	90	16,3
**Gender**		
Men	250	45,2
Women	299	54,1
Not specified	4	0,7
**Nationality**		
Spanish	511	92,4
Other	42	7,6
**Marital status**		
Single	110	19,9
Married	298	53,9
Divorced	55	9,9
Widowed	69	12,5
Other	10	1,8
Not specified	11	2
**Family composition**		
Lives alone	88	15,9
Lives with family	439	79,4
Other	15	2,7
Not specified	11	2,0
**Religion**		
Christianity	303	54,8
Islam	8	1,4
Other	21	3,8
Not specified	221	40,8
**Type of admission**		
Scheduled	166	30,0
Emergency	348	62,9
Not specified	39	7,1

Regarding gender, 54.1 % of participants were women, while 45.2 % were men. Most participants had Spanish nationality (92.4 %). In terms of marital status, more than half were married (53.9 %), while 19.9 % were single and 12.5 % were widowed.

Regarding family composition, most participants lived with their families (79.4 %), while 15.9 % lived alone. In terms of religious affiliation, 54.8 % identified as Christian, although 40.8 % did not specify their religion.

Finally, concerning the type of hospital admission, most patients were admitted through emergency services (62.9 %), while 30.0 % were hospitalized through scheduled admissions.

### 4.2 Construct validity

#### 4.2.1 Confirmatory factor analysis

The Confirmatory Factor Analysis was conducted to evaluate and validate the internal structure of the questionnaire, establishing a four-factor model for both the expectations and satisfaction scales, maintaining the factorial organization of the instrument’s original version. For parameter estimation, the least squares method was used, which is widely recommended in ordinal data analysis due to its ability to provide accurate estimates without requiring strict multivariate normality assumptions (Rial et al., 2006).

The goodness-of-fit indices for the confirmatory model, presented in Table 4, indicate an adequate fit for both scales. For the expectations scale, the values of the Comparative Fit Index (CFI= 0.905), Goodness of Fit Index (GFI= 0.979), Adjusted Goodness of Fit Index (AGFI= 0.971), Root Mean Square Residual (RMR= 0.056), Root Mean Square Residual Standardized (RMSR= 0.071), and Root Mean Square Error of Approximation (RMSEA= 0.093) suggest a stable and consistent factorial structure.

**Table 4:** Goodness of fit indices of the confirmatory model of the Spanish Version of the Impatient Dignity Scale.

Index	**Value**	
	**For expectation of dignity**	**For satisfaction with dignity**
CFI	0.905	0.936
GFI	0.979	0.995
AGFI	0.971	0.994
RMR	0.056	0.025
RMSR	0.071	0.038
RMSEA	0.093	0.078
Goodness of fit test	χ^2^ = 567.062; df= 98; *p* < 0,0001	χ^2^ = 561.476; df= 129; *p* < 0,0001
Reason for fit	χ^2^/df = 5,786	χ^2^/df = 4,353

**CFI:** Comparative Fit Index. **GFI:** Goodness of Fit Index. **AGFI:** Adjusted Goodness of Fit Index. **RMR:** Root Mean Square Residual. **RMSR:** Root Mean Standard Error Standardised. **RMSEA:** Root Mean Standard Error of Approximation.

For the satisfaction scale, the obtained values indicate an even stronger fit, with CFI= 0.936, GFI= 0.995, AGFI= 0.994, RMR= 0.025, RMSR= 0.038, and RMSEA= 0.078. Additionally, the χ²/df values were 5.786 for expectations and 4.353 for satisfaction, both within acceptable ranges for structural equation models, confirming the adequacy of the proposed theoretical model.

**Fig. 1:**
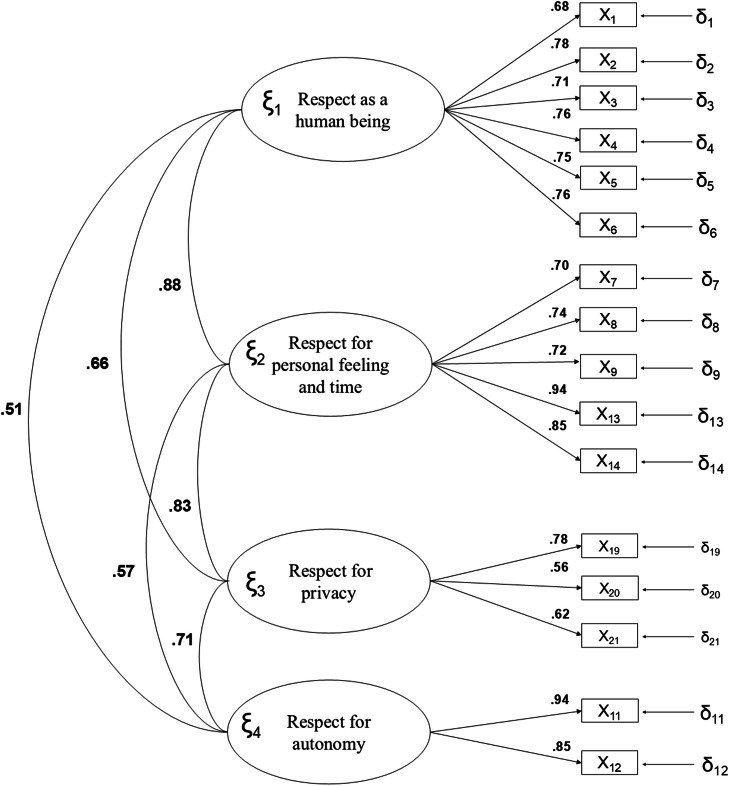
Standardized model parameters for the expectation of dignity.

**Fig. 2:**
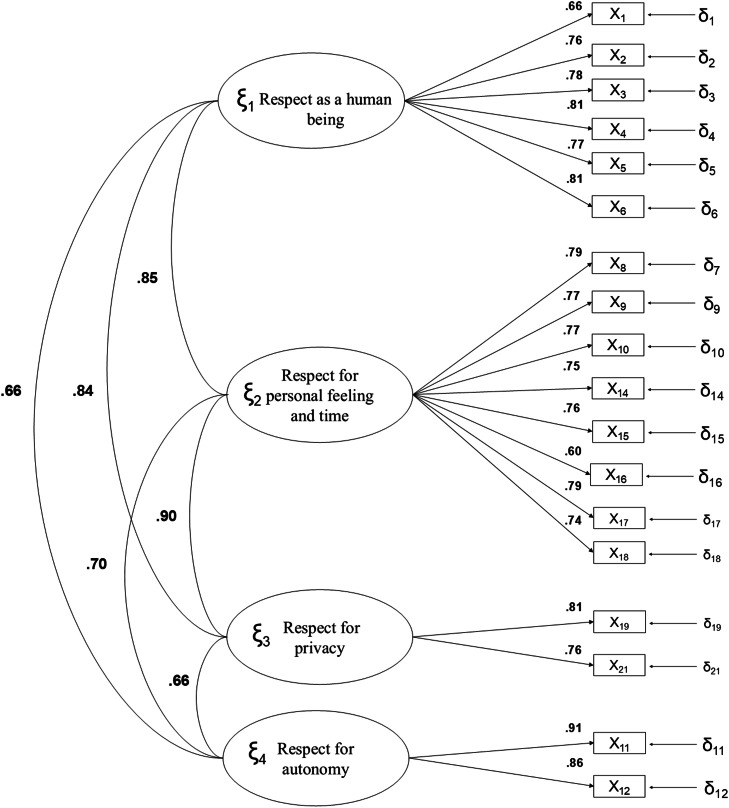
Standardized model parameters for the satisfaction with dignity.

The results of the Confirmatory Factor Analysis, represented in Fig. 1, Fig. 2, support the dimensional structure of the questionnaire in both scales. In Fig. 1, corresponding to dignity expectations, most factor loadings exceed 0.60, indicating an adequate representation of the items within each factor. Notably, the factors F1. Respect for autonomy and F2. Respect for personal feelings and time show the highest factor loadings, suggesting a greater contribution of these factors to the overall perception of dignity expectations.

In Fig. 2, representing the Confirmatory Factor Analysis results for satisfaction with dignity, a similar factorial pattern is observed. Factor loadings above 0.60 were identified for most items, with particularly high values in the F4. Respect for autonomy factor (0.91 and 0.86) and the F2. Respect for personal feelings and time factor (0.79 and 0.77).

Additionally, the correlations between factors reflect significant relationships, with a particularly high value between F2. Respect for personal feelings and time and F3. Respect for privacy (*r* = 0.90 in the satisfaction scale), suggesting substantial interdependence between these constructs.

The lowest correlations were found between F1. Respect as a human being and F4. Respect for autonomy (*r* = 0.51 in the expectations scale and *r* = 0.66 in the satisfaction scale), indicating that these domains are less related compared to other dimensions of the model. Overall, these results reinforce the structural validity of the questionnaire and support the suitability of the proposed factorial model for assessing dignity from both expectation and satisfaction perspectives.

Convergent and discriminant validity were assessed based on the results of the confirmatory factor analysis (CFA). Composite reliability (CR) values ranged from 0.711 to 0.945 for the Expectations subscale, and from 0.762 to 0.965 for the Satisfaction subscale. All values exceeded the recommended threshold of 0.70, indicating acceptable internal consistency.

Average Variance Extracted (AVE) values ranged from 0.530 to 0.905 for the Expectations subscale and from 0.620 to 0.897 for the Satisfaction subscale. These values exceeded the 0.50 criterion, supporting satisfactory convergent validity. Both CR and AVE values are reported in Table 5.

**Table 5:** Internal consistency (Cronbach’s alpha), McDonald’s omega, composite reliability, and average variance extracted for the Spanish version of the Inpatient Dignity Scale, by factor and subscale (expectation and satisfaction with dignity).

	For expectation of dignity				For satisfaction with dignity			
**Factors**	**Cronbach’s** **α**	**McDonald’s** **ω**	**Composite reliability**	**AVE**	**Cronbach’s** **α**	**McDonald’s** **ω**	**Composite reliability**	AVE
**Factor 1**	0.881	0.892	0,882	0,649	0.962	0.966	0,898	0,660
**Factor 2**	0.631	0.784	0,773	0,530	0.962	0.968	0,913	0,620
**Factor 3**	0.708	0.709	0,711	0,631	0.883	0.843	0,762	0,809
**Factor 4**	0.895	0.895	0,897	0,905	0.983	0.983	0,885	0,897
**Total Score**	**0.885**	**0.920**	**0,945**		**0.987**	0.987	**0,965**	

AVE. Average Variance Extracted.

Discriminant validity was evaluated using the Fornell–Larcker criterion. For all latent constructs, the square root of the AVE was greater than its correlations with other factors, with the exception of one borderline case in the Expectations subscale (Factor 2: √AVE= 0.728; correlation with Factor 1 = 0.732). Despite this, the overall results support the discriminant validity of both instruments.

### 4.3 Reliability

To assess the internal consistency of the questionnaire, Cronbach’s α and McDonald’s Ω coefficients were calculated both globally and for each factor in the dignity expectation and satisfaction scales. The results, presented in Table 5, indicate that the instrument demonstrates adequate reliability in both scales, with global values exceeding 0.88 for Cronbach’s α and 0.92 for McDonald’s Ω in dignity expectations, and even higher values in dignity satisfaction (α= 0.987; Ω= 0.987).

At the factorial level, in the dignity satisfaction scale, all factors showed coefficients above 0.88 in both indices, suggesting excellent reliability in measuring each dimension. In the dignity expectation scale, internal consistency was also high in most factors, with values exceeding 0.70 for both Cronbach’s α and McDonald’s Ω, except for the factor F2. Respect for personal feelings and time, where α= 0.631, which could indicate lower homogeneity among the items composing this factor. However, McDonald’s Ω coefficient for this same factor was 0.784, suggesting that this index may provide a more precise estimate by accounting for individual factor loadings.

Overall, these results support the reliability of the questionnaire and suggest that the proposed model is suitable for measuring dignity in both expectation and satisfaction terms, with a consistent and reliable internal structure across both dimensions.

## 5 Discussion

The present study aimed to conduct the cross-cultural adaptation and psychometric validation of the Inpatient Dignity Scale (Ota et al., 2019) into Spanish, assessing its factorial structure, reliability, and validity in a sample of hospitalized patients. The results confirm that the Spanish version of the Inpatient Dignity Scale is a valid and reliable instrument for measuring expectations and satisfaction with dignity in the Spanish hospital setting.

Confirmatory factor analyses supported the original four-factor structure of the instrument, maintaining the categories of respect as a human being, respect for personal feelings and time, respect for privacy, and respect for autonomy. These dimensions reflect the original concepts proposed by Griffin-Heslin (2005), on which the questionnaire is based. Lin et al. identified a similar factorial structure of dignity in their study (Ota et al., 2019). Through interviews with hospitalized patients in Taiwan, they identified six essential dimensions: the quality of care provided by nursing staff, preservation of bodily privacy, perception of control and autonomy, confidentiality of medical information, prompt response to patient needs, and recognition of respect as an individual (Lin et al., 2011).

In the original scale validated in Singapore (Ota et al., 2019), the χ²/df index was 2.32 for expectations and 2.60 for satisfaction, indicating an adequate model fit. In the Spanish version, this index was higher (5.786 for expectations and 4.353 for satisfaction), suggesting greater unexplained variability in the model. However, these values still fall within acceptable ranges in structural equation models, where values below 6 are considered adequate in health survey studies (Hu and Bentler, 1999).

Regarding incremental fit indices, in the original scale, the Comparative Fit Index (CFI) was 0.92 for expectations and 0.86 for satisfaction, while in the Spanish version, these values were 0.905 and 0.936, respectively. This suggests that the Spanish model exhibits a similar fit for dignity expectations and an improvement in the satisfaction dimension compared to the original version (Brown, 2015; Comrey, 2013; Rial et al., 2006).

Error indices, such as Root Mean Square Residual (RMSR) and Root Mean Square Error of Approximation (RMSEA), are key indicators of model fit quality in both versions. In the original scale, RMSEA was 0.09 for expectations and 0.10 for satisfaction, while in the Spanish version, these values were 0.09 and 0.07, respectively. The reduction in RMSEA values in the Spanish version suggests a lower approximation error, implying a better representation of the model in the new sample (Brown, 2015; Comrey, 2013; Rial et al., 2006).

Although the RMSEA value for the Expectations subscale (0.093) slightly exceeds the conventional cut-off of 0.08, it can be considered acceptable in the context of large-sample instrument validation. According to MacCallum et al. (1996), RMSEA values between 0.08 and 0.10 indicate a mediocre but still reasonable fit (MacCallum et al., 1996). Furthermore, RMSEA is highly sensitive to model complexity and the number of estimated parameters (Kenny et al., 2015), especially in models with multiple correlated latent variables, as is the case here. Given the strong theoretical basis of the instrument, the satisfactory results of other fit indices (CFI, GFI, AGFI), and the sample size (*N* = 553), the overall model fit is considered adequate.

Additionally, the Spanish version reports Goodness of Fit Index (GFI) values of 0.979 and 0.995, and Adjusted Goodness of Fit Index (AGFI) values of 0.971 and 0.994, reflecting an optimal model fit compared to the original version, where these indices were not reported. These findings reinforce the model's stability in the cultural adaptation of the Inpatient Dignity Scale.

A key methodological difference between the two studies is the sample size used for validation. In Singapore (Ota et al., 2019), the Inpatient Dignity Scale was validated with a sample of 363 hospitalized patients, whereas the Spanish validation included 553 participants. Since the χ² statistic is sensitive to sample size, the increase in sample size may have influenced the higher χ²/df ratio in the Spanish version. Previous studies have shown that with larger sample sizes, the χ² statistic tends to increase, potentially making the model fit appear less adequate when interpreted in isolation (Hu and Bentler, 1999).

However, joint interpretation with other fit indices, such as Confirmatory Factor Analysis and RMSEA, suggests that the factorial structure of the Spanish Inpatient Dignity Scale is robust and comparable to the original version. The improvement in RMSEA and RMSR values indicates that the Spanish adaptation presents a lower model fit error, further supporting the validity of its factorial structure.

In terms of reliability, Cronbach’s α and McDonald’s Ω coefficients reflect high internal consistency across all dimensions, with values above 0.88, suggesting that the scale accurately measures constructs related to perceived dignity during hospitalization (Cronbach, 1951; Hayes and Coutts, 2020).

Regarding internal consistency, the “Respect for personal feelings and time” dimension in the Expectations subscale yielded a Cronbach’s α of 0.631, which falls slightly below the conventional threshold of 0.70. However, this value must be interpreted with caution. First, the corresponding McDonald’s ω (0.784) and composite reliability (0.711) exceeded recommended cutoffs, suggesting acceptable internal consistency from a model-based perspective. Second, Cronbach’s α assumes tau-equivalence and is known to underestimate reliability in factors with few items or in constructs with slightly heterogeneous item content (Hayes and Coutts, 2020). In this case, the dimension consists of five items reflecting nuanced aspects of patient-provider interactions, such as the perception of being listened to, the time allocated by staff, or the degree of emotional understanding perceived by the patient. These elements may vary in relevance depending on the individual. The corresponding dimension in the Satisfaction subscale demonstrated much higher internal consistency (α= 0.883; ω= 0.843), indicating that participants were more consistent in evaluating their actual care experiences than in rating abstract expectations. These findings suggest that variability in expectations may be context dependent and influenced by prior experiences, sociocultural background, or individual preferences. Therefore, although the internal consistency of the Expectations subscale is slightly lower, it remains theoretically justifiable and statistically acceptable. Item elimination is not warranted at this stage. Nevertheless, future studies are encouraged to examine the dimensional performance in more detail, particularly in diverse clinical contexts.

The reliability coefficients in the Spanish version exceeded those reported in the original scale (Ota et al., 2019), indicating better internal stability and a more precise measurement of the evaluated constructs.

The obtained results align with previous studies emphasizing dignity as a crucial component of the hospital experience for patients. Prior research has identified that dignity perception is directly related to satisfaction with medical care and nursing care quality (Baillie, 2009; Ferri et al., 2015; Fuseini et al., 2022; Matiti and Trorey, 2008; Shojaei et al., 2023). Factors such as the hospital environment, communication, and relationships with healthcare staff (Fuseini, Rawson, et al., 2023; Lin et al., 2011; Matiti and Trorey, 2008), as well as privacy and autonomy (Ferri et al., 2015; Lin et al., 2011; Mema et al., 2025), play a key role in patients’ perception of dignity. Implementing measures that promote these factors can significantly enhance the hospital experience and improve patient well-being.

In this regard, the validation of the Spanish version of the *Inpatient Dignity Scale* (Ota et al., 2019) provides a reliable instrument for assessing these aspects within the healthcare system, which could contribute to the implementation of interventions aimed at improving patient-centered care.

### 5.1 Practical implications and clinical applicability

The validation of the Inpatient Dignity Scale in Spanish has significant implications for clinical practice and health research. Its implementation in hospitals and healthcare facilities can provide objective data on patients' perception of dignity, enabling healthcare professionals to design strategies to improve care quality, enhance communication, and foster respect in the hospital setting. Furthermore, its use in research studies may help generate evidence on the factors influencing patient dignity and their relationship with other indicators of healthcare quality.

### 5.2 Limitations and future research directions

Despite the positive results obtained, this study presents some limitations that should be considered when interpreting the findings.

First, the sample was obtained from a specific hospital setting in Spain, which may limit the generalizability of the results to other populations or healthcare systems with different characteristics. The perception of dignity during hospitalization may be influenced by cultural, structural, and organizational factors within the healthcare system. Therefore, future research should extend the validation of the Inpatient Dignity Scale in Spanish to other healthcare settings, including primary care, palliative care units, and long-term care facilities, to assess its applicability across diverse clinical contexts.

Another limitation is that the study was based on a cross-sectional design, preventing an evaluation of the instrument’s temporal stability. It is unclear whether patients’ perception of dignity remains constant or varies throughout hospitalization. Future studies could incorporate longitudinal designs, assessing the same patients at different time points during hospitalization and after discharge to analyze the reproducibility of the Inpatient Dignity Scale and its sensitivity to changes in dignity perception.

In addition, no test-retest reliability analyses were conducted, which limits the ability to assess the temporal stability of the scale. Future research should address this aspect to strengthen its use in clinical and follow-up contexts.

Additionally, this study did not explore the instrument’s discriminant and convergent validity in depth, limiting the understanding of how the Inpatient Dignity Scale relates to other measures of healthcare quality and patient well-being. Future research should include analyses comparing the Inpatient Dignity Scale with previously validated scales measuring patient satisfaction with healthcare, autonomy, and quality of life, to provide additional evidence on the instrument’s validity.

However, no external standardized measures of satisfaction or autonomy were collected in this study, which prevented any exploratory analysis through correlations with established instruments. This limitation has implications for the strength of the construct validity evidence and should be addressed in future research.

Moreover, the criterion-related validity of the instrument could not be evaluated due to the absence of an established gold standard or comparable validated instruments in Spanish specifically assessing dignity during hospitalization. This unavailability represents a limitation for assessing the scale’s predictive capacity in relation to other clinically relevant outcomes.

Finally, although the scale demonstrated good fit indices and high reliability, its factorial structure may vary depending on participants’ demographic characteristics, such as age, educational level, or health status. Future studies should conduct factorial invariance analyses to determine whether the Inpatient Dignity Scale maintains its structure across different population subgroups and validate its applicability in culturally and socioeconomically diverse populations.

## 6 Conclusions

This study provides evidence that the Spanish version of the Inpatient Dignity Scale is a valid and reliable instrument for assessing the dignity of hospitalized patients. Its factorial structure remains consistent with the original version, and its psychometric properties ensure its applicability in both research and clinical practice. Implementing the Inpatient Dignity Scale in Spanish in hospital settings will enable a better understanding of the patient experience from their own perspective, facilitating the development of strategies aimed at improving healthcare based on respect and dignity.

## Data availability

Replication data from the Spanish validation of the IPDS scale in hospitalized patients.

• Doi: https://doi.org/10.34810/data2180
• Link: https://dataverse.csuc.cat/previewurl.xhtml?token=c4d1ccca-9ede-40d0-8cab-68b496d7012a

## Funding

This study was supported by external funding from the Fundació Infermeria i Societat through the Support for Nursing Research Projects program *Ajudes a projectes de Recerca Infermera* (COIB), Number: PR-554/2022. Additionally, it also received funding from the Fundació Victor Grifols i Lucas*,* Number: PR-2023.

## Declaration of generative AI and AI-assisted technologies in the writing process

During the preparation of this work the authors used ChatGPT in order to writing process to improve the readability and language of the manuscript. After using this tool, the authors reviewed and edited the content as needed and takes full responsibility for the content of the published article.

## CRediT authorship contribution statement

**Juan Roldan-Merino:** Writing – review & editing, Writing – original draft, Visualization, Validation, Supervision, Project administration, Methodology, Funding acquisition, Formal analysis, Data curation, Conceptualization. **Carmen Jerez-Molina:** Writing – review & editing, Writing – original draft, Validation, Resources, Methodology, Conceptualization. **Olga Mestres-Soler:** Writing – review & editing, Validation, Resources, Methodology, Investigation. **Lucia Muñoz-Narbona:** Writing – review & editing, Validation, Resources, Methodology, Investigation. **Montserrat Gutiérrez-Juarez:** Writing – review & editing, Validation, Resources, Methodology, Investigation. **Ainoa Biurrun-Garrido:** Writing – review & editing, Writing – original draft, Methodology, Conceptualization. **Jéssica Gutiérrez-Martínez:** Writing – review & editing, Validation, Resources, Methodology, Investigation. **Jurema Lopez-Monreal:** Writing – review & editing, Validation, Resources, Methodology, Investigation. **Clara Expósito-Guanter:** Writing – review & editing, Validation, Resources, Methodology, Investigation. **Martí Boix-Coll:** Writing – review & editing, Validation, Resources, Methodology, Investigation. **Lucia Peñarrubia-San-Florencio:** Writing – review & editing, Validation, Resources, Methodology, Investigation. **Ramon Mir-Abellan:** Writing – review & editing, Validation, Resources, Methodology, Investigation.

## Declaration of competing interest

The authors declare that they have no known competing financial interests or personal relationships that could have appeared to influence the work reported in this paper.

## Appendix Supplementary materials

Image, application 1

Image, application 2

Image, application 3
