# Supplementary material for: Adaptation and validation of Spanish version of the Inpatient Dignity Scale in hospitalized patients: a psychometric study
Source: Int J Nurs Stud Adv. 2025 Sep 13;9:100423. doi: 10.1016/j.ijnsa.2025.100423 (PMC12489832; doi:10.1016/j.ijnsa.2025.100423)
Supplement: Supplementary file 2 [file mmc2.docx]

**IPDS Questionnaire**

| Expectations subscale | | no expectations | not very high expectations | moderately high expectations | high expectations | very high expectations |
| --- | --- | --- | --- | --- | --- | --- |
| 1 | (P/N) treat and care for me as a living human being rather than an object. | ▭ | ▭ | ▭ | ▭ | ▭ |
| 2 | (P/N) maintain eye contact with me while talking. | ▭ | ▭ | ▭ | ▭ | ▭ |
| 3 | (P/N) respect me as a human being. | ▭ | ▭ | ▭ | ▭ | ▭ |
| 4 | (P/N) listen to me attentively. | ▭ | ▭ | ▭ | ▭ | ▭ |
| 5 | (P/N) always use polite language. | ▭ | ▭ | ▭ | ▭ | ▭ |
| 6 | (P/N) are polite to my family as well as to me. | ▭ | ▭ | ▭ | ▭ | ▭ |
| 7 | (P/N) talk to me at my eye level by sitting on a chair or bending. | ▭ | ▭ | ▭ | ▭ | ▭ |
| 8 | (P/N) give my needs or expectations priority in their everyday practice. | ▭ | ▭ | ▭ | ▭ | ▭ |
| 9 | (P/N) greet me first when they see me in the hospital. | ▭ | ▭ | ▭ | ▭ | ▭ |
| 11 | (P/N) let me participate in the decision-making processes regarding my own treatment choices. | ▭ | ▭ | ▭ | ▭ | ▭ |
| 12 | (P/N) offer different choices so I can decide on my treatment. | ▭ | ▭ | ▭ | ▭ | ▭ |
| 13 | (N) of my gender give me care | ▭ | ▭ | ▭ | ▭ | ▭ |
| 14 | (P/N) understand my suffering and sympathize with me. | ▭ | ▭ | ▭ | ▭ | ▭ |
| 19 | (P/N) share my information with other members of the health-care team if necessary. | ▭ | ▭ | ▭ | ▭ | ▭ |
| 20 | (P/N) do not disclose my sensitive information, such as family issues, to health-care workers other than my own physicians and nurses. | ▭ | ▭ | ▭ | ▭ | ▭ |
| 21 | (P/N) do not collect information that is unnecessary for my medical treatment or nursing care. | ▭ | ▭ | ▭ | ▭ | ▭ |

**(N):** Nurses; **(P/N):** Physicians / Nurses

| Satisfaction subscale | | very dissatisfied | somewhat dissatisfied | moderately satisfied | satisfied | very satisfied |
| --- | --- | --- | --- | --- | --- | --- |
| 1 | (P/N) treat and care for me as a living human being rather than an object. | ▭ | ▭ | ▭ | ▭ | ▭ |
| 2 | (P/N) maintain eye contact with me while talking. | ▭ | ▭ | ▭ | ▭ | ▭ |
| 3 | (P/N) respect me as a human being. | ▭ | ▭ | ▭ | ▭ | ▭ |
| 4 | (P/N) listen to me attentively. | ▭ | ▭ | ▭ | ▭ | ▭ |
| 5 | (P/N) always use polite language. | ▭ | ▭ | ▭ | ▭ | ▭ |
| 6 | (P/N) are polite to my family as well as to me. | ▭ | ▭ | ▭ | ▭ | ▭ |
| 8 | (P/N) give my needs or expectations priority in their everyday practice. | ▭ | ▭ | ▭ | ▭ | ▭ |
| 9 | (P/N) greet me first when they see me in the hospital. | ▭ | ▭ | ▭ | ▭ | ▭ |
| 10 | (P/N) treat my pain promptly | ▭ | ▭ | ▭ | ▭ | ▭ |
| 11 | (P/N) let me participate in the decision-making processes regarding my own treatment choices. | ▭ | ▭ | ▭ | ▭ | ▭ |
| 12 | (P/N) offer different choices so I can decide on my treatment. | ▭ | ▭ | ▭ | ▭ | ▭ |
| 13 | (N) of my gender give me care | ▭ | ▭ | ▭ | ▭ | ▭ |
| 15 | (P/N) are always cheerful to me. | ▭ | ▭ | ▭ | ▭ | ▭ |
| 16 | (P/N) talk to me privately about my issues without allowing others to hear. | ▭ | ▭ | ▭ | ▭ | ▭ |
| 17 | (P/N) keep me protected with covering or clothing while providing medical treatment or nursing care. | ▭ | ▭ | ▭ | ▭ | ▭ |
| 18 | (P/N) draw the bedside curtain or shut the door to maintain privacy during medical treatment or nursing care. | ▭ | ▭ | ▭ | ▭ | ▭ |
| 19 | (P/N) share my information with other members of the health-care team if necessary. | ▭ | ▭ | ▭ | ▭ | ▭ |
| 21 | (P/N) do not collect information that is unnecessary for my medical treatment or nursing care. | ▭ | ▭ | ▭ | ▭ | ▭ |

**(N):** Nurses; **(P/N):** Physicians / Nurses
