# Supplementary material for: Adaptation and validation of Spanish version of the Inpatient Dignity Scale in hospitalized patients: a psychometric study
Source: Int J Nurs Stud Adv. 2025 Sep 13;9:100423. doi: 10.1016/j.ijnsa.2025.100423 (PMC12489832; doi:10.1016/j.ijnsa.2025.100423)
Supplement: Supplementary file 3 [file mmc3.docx]

**Cuestionario Inpatient Dignity Scale versión española (IPDS-SP)**

A continuación, encontrará frases que describen las expectativas y satisfacción de pacientes hacia las actitudes de médicos y enfermeras (M/E) o enfermeras (E) en el hospital.

Por favor, en base a su experiencia, indique el grado de satisfacción y expectativas en el cuidado marcando en una escala de 5 puntos cuáles son sus expectativas y hasta qué punto está satisfecho con las condiciones actuales.

| Expectativas | Ninguna | No muy altas | Más o menos altas | Algo altas | Muy altas |
| --- | --- | --- | --- | --- | --- |
| 1. (M/E) me tratan y me cuidan como a un ser humano y no como un objeto. | ▭ | ▭ | ▭ | ▭ | ▭ |
| 2. (M/E) mantienen contacto visual conmigo mientras me hablan. | ▭ | ▭ | ▭ | ▭ | ▭ |
| 3. (M/E) me respetan como un ser humano. | ▭ | ▭ | ▭ | ▭ | ▭ |
| 4. (M/E) me escuchan atentamente. | ▭ | ▭ | ▭ | ▭ | ▭ |
| 5. (M/E) siempre utilizan un lenguaje educado. | ▭ | ▭ | ▭ | ▭ | ▭ |
| 6. (M/E) son educados tanto con mi familia como conmigo. | ▭ | ▭ | ▭ | ▭ | ▭ |
| 7. (M/E) me hablan a la altura de los ojos aproximándose a mí. | ▭ | ▭ | ▭ | ▭ | ▭ |
| 8. (M/E) priorizan mis necesidades o expectativas en su práctica diaria. | ▭ | ▭ | ▭ | ▭ | ▭ |
| 9. (M/E) me saludan cuando me atienden. | ▭ | ▭ | ▭ | ▭ | ▭ |
| 11. (M/E) me dejan participar en los procesos de toma de decisiones sobre mis propias | ▭ | ▭ | ▭ | ▭ | ▭ |
| 12. (M/E) me ofrecen diferentes opciones para que pueda decidir mi tratamiento. | ▭ | ▭ | ▭ | ▭ | ▭ |
| 13. (E) Prefiero que enfermeras/os de mí mismo sexo me atiendan. | ▭ | ▭ | ▭ | ▭ | ▭ |
| 14. (M/E) comprenden mi sufrimiento y empatizan conmigo. | ▭ | ▭ | ▭ | ▭ | ▭ |
| 19. (M/E) comparten mi información con otros miembros del equipo sanitario si es necesario | ▭ | ▭ | ▭ | ▭ | ▭ |
| 20. (M/E) no revelan mi información sensible, como asuntos familiares, a personal sanitario que no sean mis propios médicos y enfermeras. | ▭ | ▭ | ▭ | ▭ | ▭ |
| 21. (M/E) no recogen información necesaria para mi tratamiento médico o mis cuidados de | ▭ | ▭ | ▭ | ▭ | ▭ |

(E): Enfermera; (M/E): Médico / Enfermera

| Satisfacción | Muy insatisfecho | Algo insatisfecho | Más o menos satisfecho | Algo satisfecho | Muy satisfecho |
| --- | --- | --- | --- | --- | --- |
| 1. (M/E) me tratan y me cuidan como a un ser humano y no como un objeto. | ▭ | ▭ | ▭ | ▭ | ▭ |
| 2. (M/E) mantienen contacto visual conmigo mientras me hablan. | ▭ | ▭ | ▭ | ▭ | ▭ |
| 3. (M/E) me respetan como un ser humano. | ▭ | ▭ | ▭ | ▭ | ▭ |
| 4. (M/E) me escuchan atentamente. | ▭ | ▭ | ▭ | ▭ | ▭ |
| 5. (M/E) siempre utilizan un lenguaje educado. | ▭ | ▭ | ▭ | ▭ | ▭ |
| 6. (M/E) son educados tanto con mi familia como conmigo. | ▭ | ▭ | ▭ | ▭ | ▭ |
| 8. (M/E) priorizan mis necesidades o expectativas en su práctica diaria. | ▭ | ▭ | ▭ | ▭ | ▭ |
| 9. (M/E) me saludan cuando me atienden. | ▭ | ▭ | ▭ | ▭ | ▭ |
| 10. (M/E) tratan mi dolor de manera adecuada. | ▭ | ▭ | ▭ | ▭ | ▭ |
| 11. (M/E) me dejan participar en los procesos de toma de decisiones sobre mis propias opciones de tratamiento. | ▭ | ▭ | ▭ | ▭ | ▭ |
| 12. (M/E) me ofrecen diferentes opciones para que pueda decidir mi tratamiento. | ▭ | ▭ | ▭ | ▭ | ▭ |
| 13. (M/E) comprenden mi sufrimiento y empatizan conmigo. | ▭ | ▭ | ▭ | ▭ | ▭ |
| 15. (M/E) siempre me animan. | ▭ | ▭ | ▭ | ▭ | ▭ |
| 16. (M/E) Hablan conmigo en privado sobre mis problemas sin permitir que otros lo oigan. | ▭ | ▭ | ▭ | ▭ | ▭ |
| 17. (M/E) me protegen con cobertores o ropa mientras me proporcionan tratamiento médico o cuidados de enfermería. | ▭ | ▭ | ▭ | ▭ | ▭ |
| 18. (M/E) corren la cortina de la cabecera o cierran la puerta para mantener la intimidad durante el tratamiento médico o los cuidados de enfermería. | ▭ | ▭ | ▭ | ▭ | ▭ |
| 19. (M/E) comparten mi información con otros miembros del equipo sanitario si es necesario. | ▭ | ▭ | ▭ | ▭ | ▭ |
| 21. (M/E) no recogen información necesaria para mi tratamiento médico o mis cuidados de enfermería. | ▭ | ▭ | ▭ | ▭ | ▭ |

(E): Enfermera; (M/E): Médico / Enfermera
